# Molecular dynamics simulations of heterogeneous cell membranes in response to uniaxial membrane stretches at high loading rates

**DOI:** 10.1038/s41598-017-06827-3

**Published:** 2017-08-16

**Authors:** Lili Zhang, Zesheng Zhang, John Jasa, Dongli Li, Robin O. Cleveland, Mehrdad Negahban, Antoine Jérusalem

**Affiliations:** 1 University of Oxford, Department of Engineering Science, Oxford, OX1 3PJ UK; 2 University of Nebraska-Lincoln, Department of Mechanical and Materials Engineering, Lincoln, NE 68588 USA; 3 University of Oxford, Institute of Biomedical Engineering, Oxford, OX3 7DQ UK

## Abstract

The chemobiomechanical signatures of diseased cells are often distinctively different from that of healthy cells. This mainly arises from cellular structural/compositional alterations induced by disease development or therapeutic molecules. Therapeutic shock waves have the potential to mechanically destroy diseased cells and/or increase cell membrane permeability for drug delivery. However, the biomolecular mechanisms by which shock waves interact with diseased and healthy cellular components remain largely unknown. By integrating atomistic simulations with a novel multiscale numerical framework, this work provides new biomolecular mechanistic perspectives through which many mechanosensitive cellular processes could be quantitatively characterised. Here we examine the biomechanical responses of the chosen representative membrane complexes under rapid mechanical loadings pertinent to therapeutic shock wave conditions. We find that their rupture characteristics do not exhibit significant sensitivity to the applied strain rates. Furthermore, we show that the embedded rigid inclusions markedly facilitate stretch-induced membrane disruptions while mechanically stiffening the associated complexes under the applied membrane stretches. Our results suggest that the presence of rigid molecules in cellular membranes could serve as “mechanical catalysts” to promote the mechanical destructions of the associated complexes, which, in concert with other biochemical/medical considerations, should provide beneficial information for future biomechanical-mediated therapeutics.

## Introduction

Alterations to cellular structures in many human diseases can significantly affect the chemobiomechanical properties of the cell^1^. Molecular structural modifications induced by many therapeutic macromolecules can also cause important changes to cellular biomechanical responses and thus affect disease development^2^. In particular, major structural and compositional alterations in cell membrane complexes have been directly associated with the onset, progression, and treatments of a number of human diseases^3–11^, including cancer. These structural/compositional changes are known to directly affect the cell membrane integrity, which is crucial for maintaining the intricate cell signaling and chemically-isolated intracellular environment^12^, and thus could have significant implications in mechanical trauma^13–15^ and cancer cell death/metastasis^16–19^. As a result, cell membrane complexes have emerged as novel pharmacological and mechanical targets for a number of treatments^20–22^.

Many of the hallmarks of cancer involve the alterations of fundamental cellular structures leading to the modifications of their biomechanical and biophysical properties, which in turn influence their biological functions and disease states, see reviews^1, 23, 24^. As a result, the chemobiomechanical characteristics of cancer cells are significantly different from that of healthy cells and the corresponding cellular structures have been either pharmacologically^1, 2^ or mechanically^16–19, 23, 25^ targeted for cancer treatments.

As a potential tool in cancer therapy, therapeutic shock waves target cellular membrane complexes to mechanically destroy tumour cells and/or potentiate cell membrane disruption (a process called sonoporation) to increase cell membrane permeability and facilitate drug delivery across the membrane^24, 26–31^. However, the highly complex molecular underpinnings of shock wave interactions with healthy and diseased cellular components remain largely unknown, potentially leading to undesired damage to healthy cells^32–34^. It is therefore of substantial relevance to study how changes in molecular structures, cellular biomechanical properties, and biological functions influence, and are influenced by their intricate chemobiomechanical microenvironments and external stimuli, such as shock waves. This understanding could then offer valuable insights into new biomechanical-mediated treatment design, and potentially the pathologic basis of disease and disease progression.

Previous biomechanical assays on biological cell membranes^13, 35–40^ have reported that membrane rupture occurs when the stress or strain experienced by the membrane exceeds a critical threshold. These experimental observations indicate that membrane rupture is a time-dependent phenomenon that depends on the lipid composition. Previous in silico studies of membrane damage^14, 15, 31, 41–50^ have primarily focussed on detecting and identifying membrane pore generation under a variety of loading conditions. Although it has received considerable research attention, a direct quantitative link between molecular-level biological events, such as membrane lipids dissociation, and continuum-level biomechanical concepts, such as membrane mechanoporation, is still largely lacking.

To this end, we develop here a novel multiscale numerical framework aimed at developing a quantitative bridge linking different length scales consistently with continuum mechanics concepts. This multiscale strategy offers a better fundamental understanding of the highly complex molecular mechanisms underlying cellular deformation, interaction and damage/injury, and ultimately a tool able to optimise therapeutic effects on targeted cells whilst minimising undesired damage to healthy ones.

By employing all-atom molecular dynamics (MD) simulations, we capture the molecular responses of selected representative cellular membrane complexes with different structural features characterised by distinct biomechanical properties (flexible molecules vs. rigid counterparts) undergoing rapid mechanical loadings pertinent to therapeutic shock wave conditions (see *SI* for more information). In particular, we focus on the response characteristics of the pure lipid bilayer membrane and of the lipid bilayer patches embedded with flexible molecules and rigid inclusions. The embedded flexible protein is chosen to be represented by the integrin *αIIbβ*3 transmembrane complex. The embedded rigid inclusions are artificially created by freezing the selected lipid molecules and the integrin as rigid bodies throughout the loading process. The idealised rigid lipid and rigid integrin inclusions are respectively considered as the rigid counterparts of the flexible lipids and integrin. The effects induced by these rigid inclusions on the biomechanical features of associated membrane complexes can be investigated by directly comparing the relevant responses with their counterparts.

We then extract the continuum-like deformation and stress information for the relevant cellular parts from MD simulations. The continuum-level relationships between internal stress and strain for the pure lipid bilayer membrane and the integrin transmembrane (TM) domain under uniaxial membrane stretches do not exhibit a strong loading rate sensitivity in the engineering strain rate ~10^6^–10^10^ *s* ^−1^ range. Furthermore, we find that the embedded rigid inclusions not only stiffen the associated membrane complexes, but also markedly facilitate the stretch-induced membrane disruptions. Interestingly, no significant dependence of rupture processes on the probed strain rates is observed until membrane complexes become severely fragmented.

These results indicate that the embedding of rigid molecules in the lipid bilayer membranes could serve as “mechanical catalysts” to enhance the cellular membrane disruptions induced by the applied rapid mechanical stimuli, which, in concert with other biochemical/medical approaches, potentially offers a new efficacious means to rupture diseased cells and/or potentiate sonoporation for local delivery of therapeutic macromolecules. Our findings provide new mechanistic perspectives through which many intricate mechanosensitive processes could be quantitatively and consistently examined at the continuum level, and ultimately offer valuable guidance for significant new developments in biomechanical-mediated therapeutics.

## Methods

MD simulations have emerged as a very powerful tool that has been employed to study the behavior of all kinds of microstructures by essentially solving Newton’s equations for trajectories of a system of interacting particles^51, 52^. With the rapid increase in computational power of the past three decades and the significant development of readily available simulation programs, the use of MD simulations in molecular biology has grown substantially^53–55^. One application is in the characterisation of biomechanical and biophysical properties of cellular components and quantitative description of biological processes. However, these studies require mechanically consistent methods aimed at defining continuum-level quantities, such as stress and strain, based on the atomistic information extracted from the MD simulations.

### Molecular models

All-atom molecular structures of the selected cell membrane complexes are constructed and visualised using relevant tools available in VMD^56^.

#### Pure phospholipid bilayer membrane

**Figure 1:**
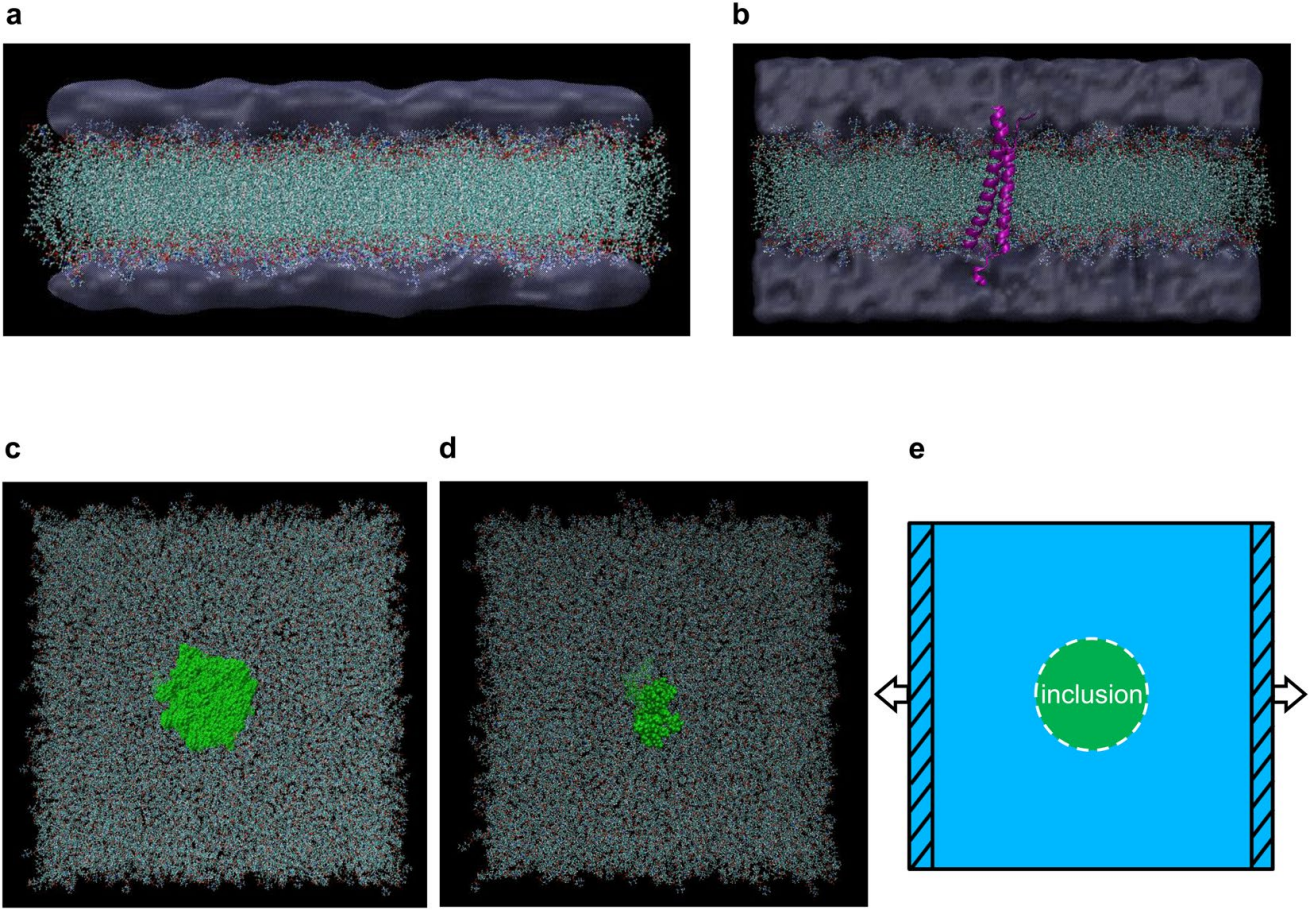
All-atom molecular models of the chosen cellular membrane complexes featuring distinct structural and biomechanical characteristics subjected to uniaxial membrane stretches in the MD simulations. The representative flexible cellular membrane complexes include: (**a**) a patch of hydrated pure 1-palmitoyl-2-oleoyl-sn-glycero-3-phosphocholine (POPC) bilayer membrane and (**b**) a hydrated lipid bilayer patch embedded with the human platelet integrin *αIIbβ*3 transmembrane complex (PDB: 2K9J). The water molecules are drawn as QuickSurf (ice blue), the flexible lipids and proteins are represented as CPK and secondary structure NewCartoon (magenta), respectively. The idealised stiffened cellular membrane complexes include: (**c**) a patch of hydrated lipid bilayer with two superposed rigid lipid parts respectively embedded in each monolayer and (**d**) a hydrated lipid bilayer patch embedded with a rigid protein (integrin *αIIbβ*3) inclusion, where the rigid inclusions are represented as VDW (green) and the water molecules are visually hidden for clarity. (**e**) schematically illustrates the implementation of uniaxial membrane stretch on each of the simulated cellular membrane complexes. Cross-sectional view: (**a** and **b**) top view: (**c**,**d** and **e**).

Figure 1a illustrates the molecular model for a patch of fully hydrated 1-palmitoyl-2-oleoyl-sn-glycero-3-phosphocholine (POPC) bilayer membrane (including 1,162 lipid molecules) with overall dimensions of about 20 × 20 × 8 *nm* ^3^ containing 298,589 atoms in total.

#### Integrin-membrane complex

As shown in Fig. 1b, one human platelet integrin *αIIbβ*3 heterodimeric transmembrane (TM) complex whose molecular structure is obtained from the Protein Data Bank (ID: 2K9J) first deposited by Lau *et al*.^57^, is embedded in the central domain of a fully hydrated POPC membrane patch (containing 899 lipid molecules) with overall dimensions of about 20 × 20 × 10 *nm* ^3^ including a total of 302,415 atoms. On circulating platelets, integrin *αIIbβ*3 is maintained in a resting conformation until platelets encounter vascular damage. It then becomes rapidly activated to mediate platelet aggregation by binding soluble ligands^58^. The dissociation of the single-pass TM receptor complex is central to the integrin bi-directional signalling processes^57^, which are known to play a critical role in integrin-mediated biological processes and various integrin-related diseases, including tumor formation and progression^59^. In the two-way signalling processes, the integrin undergoes large conformational changes and constitutes for the purpose of this study an idealised “flexible” TM protein.

#### Lipid bilayer membrane embedded with rigid inclusions

Previous biomechanical assays for cancer cells^1, 2, 60, 61^ have reported that various types of cancer cells exhibit greater stiffness due to the structural and compositional alterations of cellular components induced by the disease itself or the effects of chemotherapy. Inspired by these key observations and in an attempt to investigate how stiffened cellular parts in the membrane influence the biomechanical responses of associated membrane complexes, here we focus on two idealised but representative rigid inclusion types, which are considered as the rigid counterparts of the flexible lipid molecules and the integrin.

Figure 1c shows a patch of lipid bilayer membrane with two superposed rigid lipid parts (each with a radius of about 2 *nm*) respectively embedded in a lipid monolayer, while the remainder of the molecular model is the same as the hydrated pure lipid bilayer membrane described in Fig. 1a. Figure 1d depicts a patch of lipid bilayer embedded with a rigid protein inclusion, where the “resting conformation” of integrin *αIIbβ*3 is frozen as one rigid body throughout the process, while the remainder of the molecular system is the same as the hydrated integrin-membrane complex shown in Fig. 1b.

### MD simulations

Here, we employ all-atom MD simulations and assign CHARMM27^62^ and CHARMM36^63, 64^ force fields respectively to the POPC membrane systems and the integrin-membrane complexes. Various stages of energy minimisation and equilibration runs (following the well-established membrane-protein MD simulation protocols^65^ with appropriate modifications) are performed utilising NAMD^65^ followed by validations for each molecular model through several means in order to establish biophysically representative MD systems. The equilibrium simulation of the molecular system is completed by a run (*NPT* computation) at constant temperature (310 *K*) and constant pressure (1 *atm*) with the periodic boundary conditions applied in the three directions during which stable simulation box dimensions are achieved. The equilibrium surface area per lipid, one of the critical mechanical properties of biological membranes, is calculated for the pure POPC bilayer patch as 68.20 Å^2^ in good agreement with the experimental values of 64.3 Å^2^ and 68.3 Å^2^ reported by Kučerka *et al*.^66, 67^. The mass densities of the hydrated POPC bilayer membrane (~1.01 *g*/*cm* ^3^) and of the integrin-membrane complex (~1.01 *g*/*cm* ^3^) as well as the structural features of the lipid head groups and the backbone of protein molecules are observed to remain stable during their final phases of equilibration.

The added complexity of cellular composition/structure/geometry along with the coupling of biological cells with their 3D local environment undermine the development of a quixotic paradigm able to examine the shock wave interactions with whole cells at full atomistic resolution. Recent macroscopic experimental and computational studies^24, 68^ have shown that shock waves generate highly complex multiaxial modes, which involve complicated combinations of basic loading modes and vary during the deformation process. In an attempt to decouple and reduce the complexity, cellular deformation and damage/injury in response to simple basic loading modes have been investigated experimentally, such as uniaxial stretching of neurons^69^ and uniaxial compression of neurons^70^. We restrict here our study to uniaxial stretch, which is one of the most fundamental damaging mechanical loading modes for cells.

The subsequent production runs are carried out using LAMMPS^71^ whose initial configurations are taken from the well-equilibrated molecular systems. The MD implementation of uniaxial membrane stretch involves the following key simulation setups. The boundary condition of the MD system is switched to be non-periodic in the loading direction. In order to allow the molecular system to sufficiently adjust to the newly-switched boundary conditions, a further equilibrium simulation (*NVT* computation) is performed at constant temperature (310 *K*) and constant volume with the stable simulation box dimensions obtained from the previous *NPT* equilibration. As schematically illustrated in Fig. 1e, the two non-periodic membrane edges of each complex are then set to act as “pistons” so as to drive a transient and non-equilibrium uniaxial loading. The uniaxial stretch can be executed by pulling the two membrane “pistons” apart in the axial direction while allowing the other dimensions to freely deform. We then explore various stretching speeds in the range of 0.01–100 *m*/*s* (with estimated engineering strain rate range of ~10^6^–10^10^ *s* ^−1^) which adequately approaches the therapeutic shock wave strain rates of ~10^5^–10^7^ *s* ^−1^ (see *SI* for detailed information) so as to probe the loading rate dependence of the key biomechanical characteristics.

**Figure 2:**
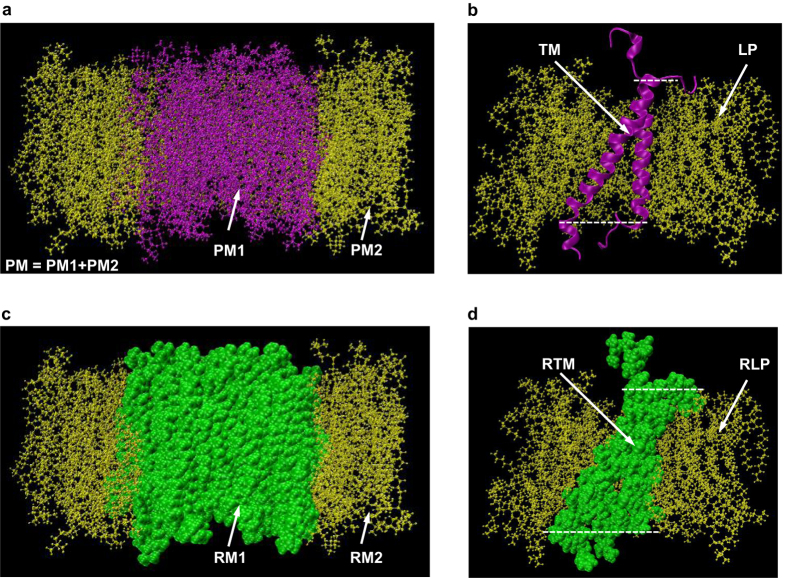
The assignment of adjacent groups consisting of interacting molecules for the selected cellular membrane complexes. The alteration of the membrane complex structural integrity can be quantitatively characterised by the evolution of the interaction between the groups. (**a**) for the pure lipid bilayer membrane, the central lipids group PM1 (with a radius of ~2 *nm*) is closely encircled by the lipids group PM2. Let group PM denote the combination of these two groups with a radius of ~3.5 *nm*. (**b**) for the integrin-membrane complex, the flexible group TM comprises the transmembrane domains of *αIIb* (residue ID 966–993) and *β*3 (residue ID 693–721) subunits, which is closely surrounded by the lipids group LP (within ~1.2 *nm* of the TM). (**c**) for the lipid bilayer patch embedded with rigid lipid inclusions, the rigid lipids group RM1 (with a radius of ~2 *nm*) is closely encompassed by the lipids group RM2 (with an outer radius of ~3.5 *nm*). (**d**) for the lipid bilayer patch embedded with rigid integrin inclusion, the rigid integrin transmembrane group RTM (*αIIb*: residue ID 966–993 and *β*3: residue ID 693–721) is closely surrounded by the lipids group RLP (within ~1.2 *nm* of the group RTM). Cross-sectional view: (**a**,**b**,**c** and **d**).

In order to set the scene for discussions on the mechanistic relationships between molecular deformations, mechanical interactions and alterations of the cellular structural integrity, groups comprising interacting molecules essential for maintaining the complex mechanical integrity are assigned for each molecular system. For the pure lipid bilayer membrane shown in Fig. 2a, group PM1 consists of the lipids in the central part of the membrane with a radius of about 2 *nm*, group PM2 is composed of the lipids in the close vicinity of group PM1 and group PM denotes the combination of both groups with a radius of about 3.5 *nm*. For the integrin-membrane complex shown in Fig. 2b, group TM represents the transmembrane domains of *αIIb* (residue ID 966–993) and *β*3 (residue ID 693–721) subunits and group LP comprises the lipids in the close neighborhood (within about 1.2 *nm*) of group TM. For the lipid bilayer membrane embedded with rigid lipid inclusions shown in Fig. 2c, group RM1 is composed of the rigid lipid parts and group RM2 denotes the lipids in the immediate vicinity of group RM1. For the lipid bilayer membrane embedded with rigid integrin inclusion shown in Fig. 2d, group RTM is composed of the transmembrane domain (*αIIb*: residue ID 966–993 and *β*3: residue ID 693–721) of the rigid integrin and group RLP comprises the lipids closely surrounding (within about 1.2 *nm*) group RTM. It should be noted that for comparison, groups PM1 and PM2 of the pure lipid bilayer membrane are assigned the same atoms as groups RM1 and RM2 of the membrane embedded with the rigid lipid inclusions, respectively. Similarly, for comparison, groups TM and LP of the integrin-membrane complex are respectively composed of the same atoms as groups RTM and RLP of the membrane embedded with the rigid integrin inclusion.

The structural alterations of the embedded inclusions and their associated surrounding lipids are illustratively highlighted in *SI* Fig. 1a–d for each molecular system subjected to stretching at rate 10^6^ *s* ^−1^. At this rate, the large deformation and subsequent dissociation of the pure lipid groups (in *SI* Fig. 1a) and of the flexible integrin subunits (in *SI* Fig. 1b) as well as the separation of the rigid lipids (in *SI* Fig. 1c) and of the rigid integrin (in *SI* Fig. 1d) from their respective neighbouring lipids are evident as expected. It is worth mentioning that the characteristics of conformational changes of these cellular complexes are similar to those observed at the other investigated rates (not shown here).

### Multiscale method to extract continuum-like quantities from MD simulations

Continuum mechanics provides a broad and fundamental framework within which macroscopic responses associated with biochemical mechanistic processes and mechanisms at the cellular and molecular levels can be consistently and quantitatively rationalised. Consequently, a number of approaches have been produced or adapted with appropriate modifications to calculate continuum-like variables based on atomistic- and molecular-level knowledge, such as the information obtained from MD simulations. Methodologies connecting the discrete atomistic or molecular quantities to the continuum descriptions of stress have attracted a long history of studies. For example, the most influential ones have been the expression developed for virial stress in 1870 by Clausius^72^, the Irving-Kirkwood-Noll procedure^73, 74^, and Hardy stress^75^ along with a variety of extensions and adaptations^76–97^. However, there are still many open questions and challenges^97^ in the overwhelming effort to link the atomistic/molecular and the continuum worlds, particularly for biological systems, where highly intricate structures are expected.

We have previously proposed a framework^86^ through which continuum-like deformation gradient and Cauchy stress can be extracted from MD simulation results for any part of the MD system. The fundamental idea is based on minimising the difference between MD measures for deformation (as a measure of the conformational changes) and traction (as a measure of the load per unit area), and their continuum counterparts. However, this work is not able to characterise the continuum-level interactions between any interacting subsets of the molecular system.

Here we are particularly interested in how biomolecular complexes mechanically interact with each other, which is critically important in numerous cellular functions and disease progressions, including cell signaling, mechanotransduction and migration, which are regulated through indirect or direct interplays between biomolecules^98–101^.

To this end, we define a continuum-like “internal stress” experienced by any set of interacting particles, and an “interaction stress” exerted on each other by any interacting parts of the MD system. The fundamental concept lies in constructing a continuum stress field that is equivalent (i.e., producing the same resultant force, moment and power) to the discrete particle forces determined by the MD simulations (see *SI* for the detailed description of the method).

It is important to note that these multiscaling concepts are independent of the MD simulation type and thus applicable to both all-atom atomistic and coarse-grained simulations. Also note that this new method is motivated by the need to quantitatively characterise continuum-level mechanical interactions between any subsets of an inhomogeneous system subjected to thermodynamically non-equilibrium and transient processes. It is thus particularly appropriate for probing shock wave interactions with cellular complexes.

The new software package that extracts the continuum-like deformation gradient, internal stress and interaction stress from MD simulation outputs is available at *UNL Digital Commons* (http://digitalcommons.unl.edu/mechengfacpub/207/) and is released under a general use license. Natively, the software directly supports LAMMPS file formats utilising the additive CHARMM force fields.

## Results

### Rate dependency in the pure lipid bilayer membrane

**Figure 3:**
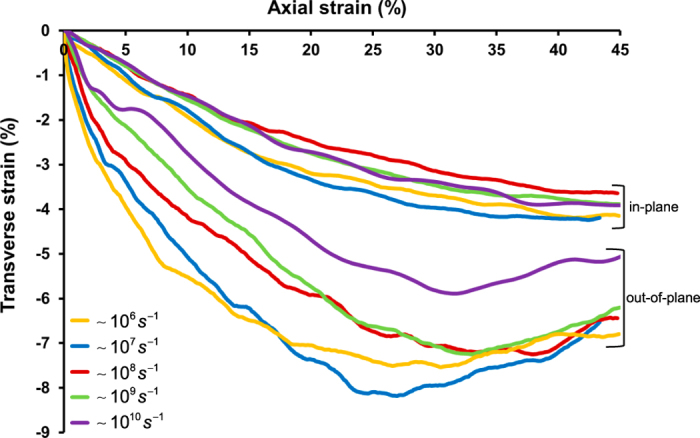
The continuum-level deformation characteristics of the cellular membrane subjected to rapid stretches (engineering strain rate range of ~10^6^–10^10^ *s* ^−1^). The Green - Lagrange strain fields are extracted from the discrete particle positions determined by the MD simulations and can be used to quantitatively assess the structural alterations of the lipid molecules in response to the mechanical loadings. The evolutions of the in-plane and out-of-plane transverse strains with the axial strain are illustrated for the central group PM of the pure lipid bilayer membrane complex.

The mechanical deformation characteristics of the pure lipid bilayer membrane in response to the applied uniaxial membrane stretches are revealed in Fig. 3, where the central group PM (as designated in Fig. 2a) is used to represent the membrane patch (so as to minimise any possible boundary effects). Using our previously-developed “MinD” method^86^, the Green-Lagrange strain fields (as a measure of the deformation) are evaluated for the probed loading rates, where the membrane in-plane and out-of-plane transverse strain evolutions resulting from the axial strain are illustrated in Fig. 3. Due to lipid membrane composition heterogeneity and boundary condition differences, the discrepancies between the in-plane and out-of-plane strain evolutions are significant. We find that the magnitude of out-of-plane strain initially increases with the axial stretch then decreases in the large deformation regime at all strain rates, indicating the dissociation of lipid molecules which might allow for the membrane thickness recovery. Note that the out-of-plane strain magnitudes at the lower strain rates (~10^6^–10^7^ *s* ^−1^) appear to be greater than those at higher rates, since slower loading process allows the molecular system relatively longer time to adjust and evolve. This feature is also slightly observable in the in-plane transverse strain evolutions.

**Figure 4:**
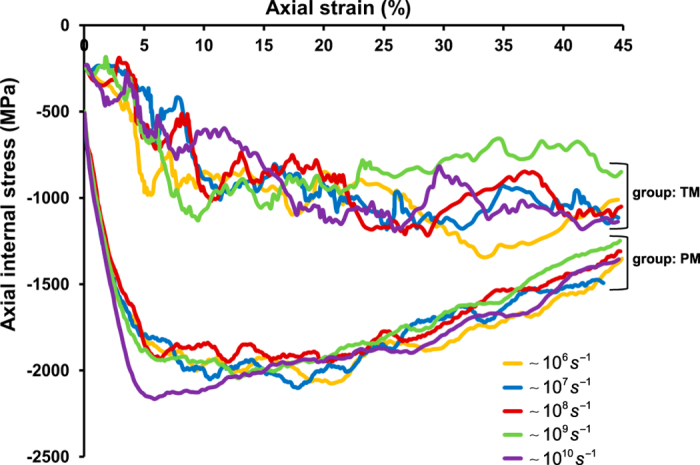
The continuum-level characterisation of how the particles within one particular group of interest mechanically influence each other under external mechanical stimuli. The evolutions of the surface-averaged internal stresses with the axial strains at a wide range of engineering strain rates (~10^6^–10^10^ *s* ^−1^) are illustrated for the chosen flexible cellular membrane complexes: the central group PM of the pure POPC bilayer membrane and the TM domain of integrin *αIIbβ*3 embedded in a patch of hydrated lipid bilayer.

Furthermore, we use the proposed internal stress (see *SI* Eq. (45)) as a continuum-level measure of how particles within a select group mechanically interact with each other. The evolution of the surface-averaged internal stress (see *SI* Eq. (46)) with the axial strain is shown in Fig. 4 for the group PM at different strain rates. No strong trend of loading rate dependence is observed, except that the magnitude of the yield stress corresponding to the highest strain rate (~10^10^ *s* ^−1^) is slightly larger than the others.

### Rate dependency in the transmembrane (TM) domain of integrin *αIIbβ*3

The biomechanical response of the integrin TM domain (see Fig. 2b) is probed by estimating how its internal stress evolves with the applied membrane stretch. As depicted in Fig. 4, the evolution of the surface-averaged internal stress (see *SI* Eq. (46)) with the axial strain for the group TM indicates that the heterodimeric TM domain behaves mechanically softer than the pure lipid bilayer membrane patch, possibly due to its single transmembrane alpha-helices structural features. It should be noted that the response of the integrin TM domain is expected to exhibit complex fluctuations as a result of its structural and compositional characteristics, while no significant trend of sensitivity to the stretching rates is observed.

### Rigid inclusions mechanically stiffen the associated lipid bilayer complexes and facilitate membrane disruptions

**Figure 5:**
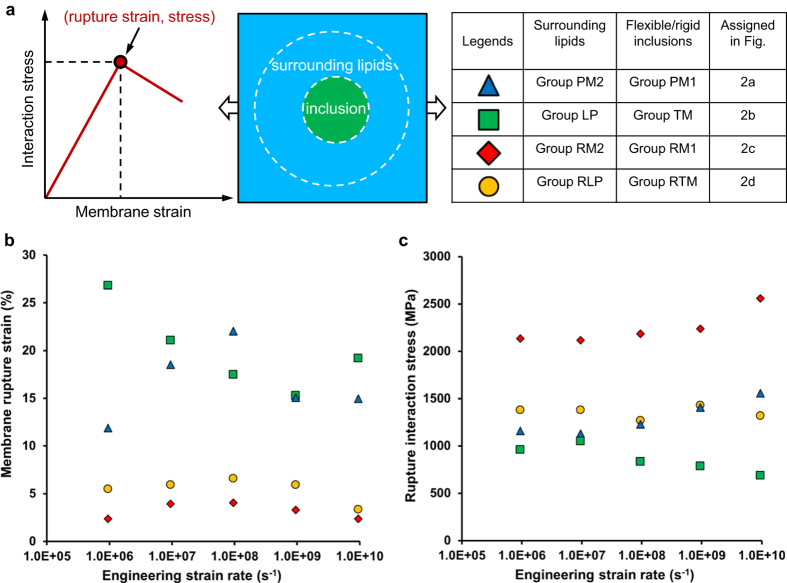
The continuum-level characterisation of how the mechanical interaction between two interacting groups evolves with the application of external mechanical stimuli. (**a**) schematically describes the general trends of the evolution of the surface-averaged interaction stress (from the surrounding lipid molecules on the embedded inclusions) against the applied membrane strain, through which the membrane rupture signatures (rupture strain and stress) can be identified. The groups designated as the flexible/rigid inclusions and the associated surrounding lipids are listed for the chosen cellular membrane complexes. (**b**) and (**c**) reveal the membrane rupture strains and the corresponding surface-averaged interaction stresses, respectively, for the biomolecular systems undergoing uniaxial membrane stretch at a broad range of loading rates (~10^6^–10^10^ *s* ^−1^). The blue colour denotes the rupture characteristics involving groups PM1 and PM2 of the pure lipid bilayer membrane. Similarly, the green, red and orange colours represent the stretch-induced membrane complex rupture features for the integrin-membrane complex, the lipid bilayer embedded with rigid lipid inclusions and the lipid bilayer embedded with rigid integrin inclusion, respectively.

We use the proposed interaction stress (see *SI* Eq. (47)) as a continuum-level mechanical measure to describe the interplay between any interacting biomolecular complexes and thus quantify the alterations of their mechanical integrity in response to the applied mechanical stimuli. Figure 5a schematically illustrates the general features of how the surface-averaged interaction stress (see *SI* Eq. (48)) from the surrounding lipid molecules on the embedded inclusions evolves with the applied membrane stretch. The evolutions of axial surface-averaged interaction stresses with the axial membrane strains applied at various loading rates are shown in *SI* Fig. 1e and f for the pure lipid bilayer and the integrin-membrane complex, respectively, together with their rigid counterparts. It is apparent that interactions with the flexible inclusions involve more complex deformations and structural adjustments, as their interaction stresses exhibit more fluctuations over the course of loadings. The axial component of the surface-averaged interaction stress initially increases with the axial strain experienced by the surrounding lipids until a certain critical strain is reached, above which the stress decreases with further membrane stretching, indicating the initiation of integrity failure between the surrounding lipids and the associated inclusions. This critical strain and its corresponding surface-averaged interaction stress are respectively termed rupture membrane strain and rupture stress, and presented in Fig. 5b and c for the selected membrane complexes (i.e., the pure lipid bilayer membrane, the integrin-membrane complex, and the lipid bilayer patches embedded with the rigid inclusions). The membrane areal strains at rupture and the corresponding rupture tensions are respectively shown in *SI* Fig. 2a and b, where the areal strain of the surrounding lipids is calculated as the area change divided by the initial area, and the membrane tension is estimated as the product of the axial component of the interaction stress with the approximate bilayer thickness (~5 *nm*). In spite of the fast loading rates and the idealised representations of cell membranes, the present membrane areal strains at rupture are in near quantitative agreement with experimental measurements of cancer cells rupture at ~5%^18^ due to stretching and of lipid bilayers rupture at ~1.2–5.1%^35, 36^ determined by micropipette aspiration. On the other hand, the estimated rupture tensions (~3435.00–12799.80 *mN/m*) are significantly greater than the tensile strengths (~3.8–5.1 *mN*/*m* ^102^; ~3–10 *mN*/*m* ^36^; ~1–25 *mN*/*m* ^38^; ~2.3–30.9 *mN*/*m* ^35^) measured in experiments at much lower loading rates. The discrepancy could be attributed to the fact that the rupture strength of biomembranes depends prominently on lipid compositions and the time frame (or loading rate) for breakage^38^. Another source of the disparity is believed to be the existence of precursor defects^38^ (such as pre-pores) in the macroscopic bilayer membranes, which could significantly lower the experimentally determined membrane rupture strength and lead to distinct membrane failure modes from the ones we observe in the MD simulations. It is finally noteworthy that the present rupture tensions are approximated based on the interaction stresses induced by the surrounding lipid molecules on the embedded inclusions, and therefore they reflect fundamentally different mechanistic perspectives from those measured experimentally at the macroscopic level.

We find that the rupture characteristics do not exhibit a significant sensitivity to the applied strain rates; however, the embedded rigid inclusions (i.e., the rigid lipid and rigid integrin inclusions) exert notable effects on the alterations of the associated membrane complex structural/mechanical integrity. When compared with the pure lipid bilayer membrane, the lipid bilayer patch embedded with the rigid lipid inclusions appears to be disrupted at much smaller strains but with greater interaction stresses, indicating that the rigid inclusions markedly facilitate the stretch-induced membrane complex disruptions, while stiffening the associated membrane complex under the application of the probed loading mode. Similar rupture characteristics can also be observed when comparing the integrin-membrane complex with its rigid counterpart (i.e., the lipid bilayer patch embedded with the rigid integrin inclusion). The weak loading rate dependence up to rupture in the studied range indicates that the membrane complexes respond in a non-diffusive quasi-elastic regime up to failure. It must be emphasised that a similar behaviour of lipid membranes has been reported in experiments^35, 38^ at much lower loading rates.

## Discussion

The past several decades have seen major breakthroughs in structural biology, where a number of atomic-resolution static structures for various proteins and other biomolecules have been determined through crystallography and other biophysical techniques^54^. Atomic-level biomolecular simulations have been an active field of research for decades, and, with the continuing improvements in computer hardware, software, and simulation methodologies, have emerged as a powerful tool for the study of biomolecular dynamics at spatiotemporal scales that are difficult to access experimentally^54^. Such experimental and computational progress has provided extraordinary opportunities for examining a wide range of fundamental biomolecular processes pertaining to physiology, pathology and therapeutics in full atomic detail. More specifically, a number of key biological discoveries^54^, such as conformational transitions in proteins, protein folding, transport across cell membranes, and the binding of ligands to their target proteins, have been made through biomolecular simulations whose applicability is also rapidly expanding in the design/development of therapeutics^25, 31, 43, 54^. The scalability limitations of these atomistic-level advances have highlighted the critical need for new methodologies aimed at linking the biophysical information of interest across different length scales so as to provide insights into the microscale mechanisms underlying the sophisticated biological phenomena involved in diseases and treatments.

To this end, this study has presented a novel multiscale computational framework within which the discrete atomic-level details obtained from MD simulations are quantitatively characterised at the continuum level in a manner consistent with classical continuum mechanics concepts. The biomolecular structural alterations of the selected representative cellular components are captured in full atomic detail by utilising all-atom MD simulations, and their corresponding continuum-level biomechanical responses (i.e., deformation, internal stress and interaction stress) are quantified via the proposed multiscale methods. Here we focus on the biomechanical signatures (i.e., deformation characteristics, loading rate dependence and membrane rupture characteristics) of the lipid bilayer membrane complexes given their ubiquitous presence and strong pathophysiological implications in human diseases and therapeutics. In particular, the simulated systems include pure POPC bilayer membranes and lipid bilayer patches embedded with flexible proteins (i.e., integrin *αIIbβ*3) and rigid inclusions (i.e., the rigid integrin and rigid lipid inclusions), which serve as idealised representations for stiffened biomolecular complexes in cell membranes induced by certain types of cancers or drug molecules^1, 2, 60, 61^. Uniaxial membrane stretches are applied on each membrane complex with a broad range of loading rates (engineering strain rate ~10^6^–10^10^ *s* ^−1^) which adequately approach the therapeutic shock wave strain rates found in the range of ~10^5^–10^7^ *s* ^−1^.

The in-plane transversal strain and the out-of-plane thickness alteration (Fig. 3) of the pure lipid bilayer membrane are revealed to evolve distinctly with the axial membrane stretch as a result of the lipid membrane composition heterogeneity and boundary condition differences, particularly in the large deformation regime where the dissociation of lipid molecules is initiated. The continuum-level internal stress vs. strain relationship suggests that the responses of the pure lipid bilayer membrane and the TM domain of integrin *αIIbβ*3 (Fig. 4) are nearly independent of the applied stretching rates. It also indicates that the integrin heterodimeric TM domain (composed of single transmembrane alpha-helices) appears to behave mechanically softer than the pure lipid bilayer patch in response to the probed loading mode. It should be noted that these mechanistic characteristics revealed through our multiscale computational framework are often difficult to be examined in a well-controlled manner by recourse to experiments alone due to the spatial and temporal limitations.

Next, we quantify how the rigid inclusions embedded in cellular membranes influence the biomechanical responses of the associated membrane complexes undergoing rapid mechanical loadings, which is achieved by drawing direct comparisons with their flexible counterparts. The rupture characteristics (rupture membrane strain in Fig. 5b and rupture stress in Fig. 5c) of each membrane complex are determined through the evolution of the continuum-level interaction stress with the membrane strain, with no significant rate dependence observed at the probed strain rates. We find that the presence of rigid molecules (i.e., the rigid lipid and rigid integrin inclusions) in the lipid bilayer membranes markedly facilitate the stretch-induced membrane disruptions, while mechanically stiffening the associated membrane complexes. These biomolecular mechanistic observations in concert with other biochemical/medical considerations offer great potential for significant new developments in biomechancial manipulation of the targeted cellular complexes so as to destroy the diseased cells and/or potentiate cell membrane sonoporation for local delivery of therapeutic molecules. We finally postulate that the drastic changes of cellular structures and compositions in some specific cancers (or under chemotherapy) could naturally offer avenues for shock wave therapy which could be tuned to exclusively rupture cancer cells.

It is important to note that there are several limitations in our study, which should be addressed by future investigations employing a similar multiscaling framework. First, all-atom MD simulations of biological systems are computationally demanding, thus placing restrictions on the achievable spatial and temporal scales. A variety of techniques (such as coarse-graining and enhanced sampling, see review^103^) can be utilised in combination with MD simulations within the presented computational framework to probe biological events that take place on timescales that remain inaccessible to direct all-atom MD simulations. Next, the complexity of biological structures is further compounded by the structural and compositional changes induced by the disease processes and drug effects, which pose additional challenges in determining the atomic-resolution crystallographic structures of such biological macromolecules and simulating their critical biochemical processes accurately. It is important to understand that the embedded rigid molecules (rigid integrin and rigid lipid inclusions) presented here only serve as the idealised representations for the highly intricate, actual cellular complexes involved in certain disease states. Albeit simplistic, these representative biomolecular models may provide a quantitative picture of the distinct biomechanical signatures of the normal and the diseased cellular components in response to the therapeutic shock waves and potentially offer possible avenues for cancer therapy. Lastly, it should be noted that our current study focusses on quantitatively characterising the key biomechanical properties (e.g., deformation characteristics, rate sensitivity, biomolecular interaction and rupture features) of the cellular membrane complexes subjected to a monotonic constant-rate stretching phase, rather than more complex multiaxial loading states implicated in the shock wave profile (see *SI* Fig. 3b) comprising a short duration of positive pressure followed by a relatively longer duration of negative pressure at varying loading rates. Other basic loading modes (e.g., biaxial tension, compression and shear) at a broader range of rates exerted on other important biomolecular complexes featuring various structural/compositional characteristics are currently being investigated and will provide additional insight into the full effect of therapeutic shock waves on the diseased/healthy cellular components. This work provides a foundation for quantitatively examining the sophisticated biological processes from molecular mechanistic perspectives. Future studies should expand the current investigation to evaluate how the essential cellular functions and regulations, such as gating mechanisms of ion channels, drug binding efficacy, cell mechanotransduction and cellular signaling are affected after the exposure to shock wave therapy, thus establishing comprehensive connections between biomolecular/cellular mechanics and biological cell functions in human health and disease.

## Electronic supplementary material

Molecular dynamics simulations of heterogeneous cell membranes in response to uniaxial membrane stretches at high loading rates
