# Supplementary material for: Molecular dynamics simulations of heterogeneous cell membranes in response to uniaxial membrane stretches at high loading rates
Source: Sci Rep. 2017 Aug 16;7:8316. doi: 10.1038/s41598-017-06827-3 (PMC5559491; doi:10.1038/s41598-017-06827-3)
Supplement: Supplementary file 1 — Molecular dynamics simulations of heterogeneous cell membranes in response to uniaxial membrane stretches at high loading rates [file 41598_2017_6827_MOESM1_ESM.pdf]

SI: Molecular dynamics simulations of heterogeneous cell membranes in response to uniaxial membrane stretches at high loading rates

Lili Zhang[1], Zesheng Zhang[2], John Jasa[2], Dongli Li[3], Robin O. Cleveland[3], Mehrdad Negahban[2] and Antoine Jérusalem[1]

[1] University of Oxford, Department of Engineering Science, Oxford, OX1 3PJ, UK

[2] University of Nebraska-Lincoln, Department of Mechanical and Materials Engineering, Lincoln, NE 68588, USA

[3] University of Oxford, Institute of Biomedical Engineering, Oxford, OX3 7DQ, UK

1 Evaluating the strain rates pertinent to therapeutic shock waves via finite element simulations

The stretching rates applied as the input boundary conditions in the atomistic molecular dynamics (MD) simulations are informed by the strain rates calculated in the finite element (FE) simulations of shock wave interactions with a whole cell. As illustrated in *SI* Fig.3a, a 3D continuum FE model has been developed with a combined equation of state (for the volumetric response) and hyper-viscoelastic (for the deviatoric response) material framework to simulate single cell deformation in response to therapeutic shock waves. An analytical shock wave profile measured at the lithotripter focus (see *SI* Fig.3b) is applied at the top surface of the FE model and assumed to propagate as a plane wave in a small volume of medium. This numerical model is calibrated and validated against the experimental observations of single cell deformation under the action of shock waves using ultra-high speed imaging (see Ref.[2] for more details). The FE simulation results show that the tensile strain rates experienced by the cortical membrane are between about 10^5 and $10^7 s^{-1}$, which motivate the loading rates used in the MD simulations reported here.

2 Multiscale method to extract continuum-like quantities from MD simulations

Here, we describe the proposed method aimed at evaluating the continuum-level characteristics from the MD-determined atomistic details in a quantitative manner consistent with classical continuum mechanics concepts. In order to establish the connections between the discrete and the continuum worlds, a brief introduction of the concept of a continuum body and its mechanics is presented first to set the stage for subsequent derivations. This is followed by a brief description of a typical MD system and the steps to construct its equivalent homogeneous continuum body and evaluate the corresponding continuum velocity field. The fundamental concept of our multiscale approach is illustrated next by considering a single force exerted on a particle and developing an equivalent continuous traction distributed over the surface of a continuum body. The traction is taken to produce exactly the same resultant force, moment and power for a given velocity field as the force does on the particle. This idea is then extended via the superposition principle to investigate different types of forces applied on any set of particles, and the continuum-level internal and interaction stresses are respectively defined within this multiscaling framework.

2.1 Continuum body

A continuum body is defined as an enclosed continuous distribution of mass occupying a volume V with surface S. Each material point in this body is described by its position vector $\mathbf{x}$, velocity $\mathbf{v}$, acceleration $\mathbf{a}$ and mass density ρ. The total mass m and position of the centre of mass $\mathbf{x}_{CM}$ of the continuum body are given, respectively, by

$$m = \int_V \rho dV, \tag{1}$$

$$\mathbf{x}_{CM} = \frac{1}{m} \int_V \mathbf{x} \rho dV. \tag{2}$$

Note that for a homogeneous continuum body, its centroid of volume coincides with its centre of mass.

The mass moment of inertia tensor $\bar{\mathbf{I}}_m$ relative to its centre of mass is defined by

$$\bar{\mathbf{I}}_m = \int_V \left[(\boldsymbol{\rho} \cdot \boldsymbol{\rho}) \, \mathbf{1} - \boldsymbol{\rho} \otimes \boldsymbol{\rho} \right] \rho dV, \tag{3}$$

where "$\mathbf{1}$" denotes the second order identity tensor, "$\cdot$" represents the dot product, "$\otimes$" denotes the tensor product, and $\boldsymbol{\rho} = \mathbf{x} - \mathbf{x}_{CM}$ represents the relative position of material point at $\mathbf{x}$ with respect to the centre of mass CM.

When a traction $\mathbf{t}^{(n)}$ is exerted on the surface S with normal $\mathbf{n}$, the total load $\mathbf{f}$, the total moment $\mathbf{C}$ about CM, and the total power $\dot{W}$ produced by the traction can be respectively calculated from

$$\mathbf{f} = \int_S \mathbf{t}^{(n)} dS, \tag{4}$$

$$\mathbf{C} = \int_S \boldsymbol{\rho} \times \mathbf{t}^{(n)} dS, \tag{5}$$

$$\dot{W} = \int_S \mathbf{v} \cdot \mathbf{t}^{(n)} dS, \tag{6}$$

where "$\times$" denotes the cross product.

2.2 Discrete MD system and its equivalent homogeneous continuum body

SI Fig.4a schematically depicts a typical MD system consisting of discrete particles. Group Ω is composed of the particles within any subsystem that is of particular interest, e.g., a specific subdomain of a protein. Subscript i is used to denote the quantities associated with particle i ($i \in \Omega$). The total mass m and position of the centre of mass $\mathbf{r}_{CM}$ of the group are given, respectively, by

$$m = \sum_{i \in \Omega} m_i, \tag{7}$$

$$\mathbf{r}_{CM} = \frac{1}{m} \sum_{i \in \Omega} m_i \mathbf{r}_i, \tag{8}$$

where m_i and $\mathbf{r}_i$ denote the mass and the position vector of particle i, respectively. Let $\mathbf{v}_{CM}$ denote the velocity of the centre of mass calculated from

$$\mathbf{v}_{CM} = \frac{1}{m} \sum_{i \in \Omega} m_i \mathbf{v}_i, \tag{9}$$

where $\mathbf{v}_i$ is the velocity of particle i.

The relative position of particle i with respect to the centre of mass CM is represented by $\boldsymbol{\rho}_i$ and given by

$$\boldsymbol{\rho}_i = \mathbf{r}_i - \mathbf{r}_{CM}. \tag{10}$$

The mass moment of inertia tensor $\bar{\mathbf{I}}_m$ of the group is defined as

$$\bar{\mathbf{I}}_m = \sum_{i\in\Omega} m_i \left[\left(\boldsymbol{\rho}_i \cdot \boldsymbol{\rho}_i \right) \mathbf{1} - \boldsymbol{\rho}_i \otimes \boldsymbol{\rho}_i \right]. \tag{11}$$

The eigenvalues and the corresponding normalised eigenvectors of $\bar{\mathbf{I}}_m$ are respectively denoted by $\bar{I}_1, \bar{I}_2, \bar{I}_3$ and $\mathbf{x}^{(1)}, \mathbf{x}^{(2)}, \mathbf{x}^{(3)}$. The spectral representation of $\bar{\mathbf{I}}_m$ can then be written as

$$\bar{\mathbf{I}}_m = \bar{I}_1 \mathbf{x}^{(1)} \otimes \mathbf{x}^{(1)} + \bar{I}_2 \mathbf{x}^{(2)} \otimes \mathbf{x}^{(2)} + \bar{I}_3 \mathbf{x}^{(3)} \otimes \mathbf{x}^{(3)}. \tag{12}$$

We have shown previously [4] that a solid ellipsoid of constant density can be constructed whose centre of mass, total mass and mass moment of inertia are respectively the same as those of the discrete particle system. The principal axes of the ellipsoid are chosen to align with $\mathbf{x}^{(1)}, \mathbf{x}^{(2)}, \mathbf{x}^{(3)}$ and the associated principal radii are denoted by $\alpha_1, \alpha_2, \alpha_3$. The mass moment of inertia of the solid ellipsoid can then be written as

$$\bar{\mathbf{I}}_m = \frac{m}{5} \left[\left(\alpha_2^2 + \alpha_3^2 \right) \mathbf{x}^{(1)} \otimes \mathbf{x}^{(1)} + \left(\alpha_1^2 + \alpha_3^2 \right) \mathbf{x}^{(2)} \otimes \mathbf{x}^{(2)} + \left(\alpha_1^2 + \alpha_2^2 \right) \mathbf{x}^{(3)} \otimes \mathbf{x}^{(3)} \right], \tag{13}$$

which is required to be equal to that of group Ω (see *SI* Eq.(12)). The principal radii $(\alpha_1, \alpha_2, \alpha_3)$ of the ellipsoid can be determined in terms of the eigenvalues $\left(\bar{I}_1, \bar{I}_2, \bar{I}_3 \right)$ of the particle group via the following relation

$$\left\{ \begin{array}{c} \alpha_1 \\ \alpha_2 \\ \alpha_3 \end{array} \right\} = \sqrt{\frac{5}{2m}} \left\{ \begin{array}{c} \sqrt{-\bar{I}_1 + \bar{I}_2 + \bar{I}_3} \\ \sqrt{\bar{I}_1 - \bar{I}_2 + \bar{I}_3} \\ \sqrt{\bar{I}_1 + \bar{I}_2 - \bar{I}_3} \end{array} \right\}. \tag{14}$$

The volume bounded by the ellipsoid is

$$V = \frac{4\pi}{3} \alpha_1 \alpha_2 \alpha_3. \tag{15}$$

The idea of constructing a homogeneous continuum body that is equivalent to the discrete particle system is not restricted to the ellipsoidal shape presented here. One may select any continuum shape primarily depending on the structural features of the discrete system.

2.3 Evaluating the continuum velocity field

This section focusses on establishing the relation between the particle velocities of the discrete system and the velocity field of the equivalent homogeneous continuum body. To this end, the minimisation method [4] we have previously proposed is employed here and briefly summarised in the following.

We assume that the velocity of the centre of mass of the homogeneous continuum body is identical to that of the discrete particle group (see *SI* Eq.(9)), which can be directly determined from the MD simulations. The velocity $\mathbf{v}$ of the material point at $\mathbf{x}$ in the continuum body can be written via a Taylor series expansion around its centre of mass at $\mathbf{r}_{CM}$ as

$$\mathbf{v} = \mathbf{v}_{CM} + \mathbf{L}\boldsymbol{\rho} + \frac{1}{2}\mathbf{L}^{(2)} : (\boldsymbol{\rho} \otimes \boldsymbol{\rho}) + \frac{1}{3!}\mathbf{L}^{(3)} \vdots (\boldsymbol{\rho} \otimes \boldsymbol{\rho} \otimes \boldsymbol{\rho}) + ..., \tag{16}$$

where $\boldsymbol{\rho} = \mathbf{x} - \mathbf{r}_{CM}$, and $\mathbf{L}$ and $\mathbf{L}^{(n)}$ respectively denote the first order and the nth order velocity gradient evaluated at CM. The tensor operators ":" and "$\vdots$" respectively denote the double and triple dot products.

In this study we consider a linear velocity field $\tilde{\mathbf{v}} = \mathbf{v}_{CM} + \mathbf{L}\boldsymbol{\rho}$, i.e., the expansion includes up to the first order velocity gradient having the higher order terms truncated. As might be expected, by introducing the location of any particle into the continuum velocity field one obtains a velocity that is not exactly the same as the actual particle velocity. Let us denote this difference (error) for particle i as $\boldsymbol{\Delta}_i$ so that

$$\mathbf{v}_i = \mathbf{v}_{CM} + \mathbf{L}\boldsymbol{\rho}_i + \boldsymbol{\Delta}_i. \tag{17}$$

The total error for the particle system can be evaluated by introducing an overall error function R that sums up the error of all the individual particles

$$R = \frac{1}{2}\sum_{i \in \Omega} m_i \, (\boldsymbol{\Delta}_i \cdot \boldsymbol{\Delta}_i), \tag{18}$$

where the particle mass m_i is used as the weighting factor associated with particle i.

Our task is to select a velocity gradient $\mathbf{L}$ that minimises the overall error R. As is common in data regression, the optimal fit is the one for which the variation in R is zero for all possible variations of $\mathbf{L}$. This corresponds to finding the stationary point of the overall error function. The variation in R can be written as

$$\delta R = \partial_{\mathbf{L}}(R) : \delta \mathbf{L}, \tag{19}$$

where $\delta\mathbf{L}$ denotes the variation in $\mathbf{L}$. At the stationary point, δR has to be equal to zero for all possible $\delta\mathbf{L}$, which requires that $\partial_{\mathbf{L}}(R) = \mathbf{0}$. This results in

$$-\sum_{i\in\Omega} m_i \left[\mathbf{v}_i - (\mathbf{v}_{CM} + \mathbf{L}\boldsymbol{\rho}_i)\right] \otimes \boldsymbol{\rho}_i = \mathbf{0}, \tag{20}$$

from which the first order velocity gradient $\mathbf{L}$ can be solved as

$$\mathbf{L} = \left\{\sum_{i\in\Omega} \left[m_i \left(\mathbf{v}_i - \mathbf{v}_{CM}\right) \otimes \boldsymbol{\rho}_i\right]\right\} \left[\sum_{i\in\Omega} \left(m_i \boldsymbol{\rho}_i \otimes \boldsymbol{\rho}_i\right)\right]^{-1}. \tag{21}$$

Let $\tilde{\mathbf{v}}_i$ denote the first order approximation of the actual velocity $\mathbf{v}_i$ for particle i, which can now, using *SI* Eq.(21), be calculated from

$$\tilde{\mathbf{v}}_i = \mathbf{v}_{CM} + \mathbf{L}\boldsymbol{\rho}_i. \tag{22}$$

2.4 Evaluating the continuum traction and stress

This section focusses on establishing the connection between the discrete load applied on the particle and the corresponding continuous traction and stress exerted on the equivalent homogeneous continuum body. As shown in *SI* Fig.4b, let $\mathbf{f}_j$ denote a single load applied on particle j ($j \in \Omega$) at the relative position $\boldsymbol{\rho}_j$ with respect to the centre of mass CM of the discrete particle group Ω. An equivalent homogeneous continuum body with its centre of mass at CM (i.e., coinciding with the centre of mass of the particle group Ω) occupying the volume V enclosed by its surface S can be constructed. The aim is to find a continuous traction distribution $\mathbf{t}^{(n)}$ over the continuum surface with normal $\mathbf{n}$ that generates exactly the same resultant force, moment and power as the load $\mathbf{f}_j$ produces on particle j.

The traction vector $\mathbf{t}^{(n)}$ can be written in an orthogonal basis (formed by basis vectors $\mathbf{a}$, $\mathbf{b}$ and $\mathbf{f}_j$) as

$$\mathbf{t}^{(n)} = \mu\mathbf{f}_j + \eta\mathbf{a} + \gamma\mathbf{b}, \tag{23}$$

where the vectors $\mathbf{a}$ and $\mathbf{b}$ are selected to be any two non-zero orthogonal vectors (i.e., $\mathbf{a}\cdot\mathbf{b} = 0$) in the plane perpendicular to the load $\mathbf{f}_j$ (i.e., $\mathbf{f}_j \cdot \mathbf{a} = 0$ and $\mathbf{f}_j \cdot \mathbf{b} = 0$), and μ, η and γ are the unknown scalar functions of space.

Let us first examine the requirement that the resultant force generated by the continuum trac-

tion (see *SI* Eq.(4)) has to be the same as that of the load $\mathbf{f}_j$, which gives

$$\int_S \mathbf{t}^{(n)} dS = \mathbf{f}_j. \tag{24}$$

Substituting *SI* Eq.(23) into this relation results in

$$\left(\int_S \eta dS\right)\mathbf{a} + \left(\int_S \gamma dS\right)\mathbf{b} = \left(1 - \int_S \mu dS\right)\mathbf{f}_j. \tag{25}$$

After taking the orthogonality of $\mathbf{f}_j$, $\mathbf{a}$ and $\mathbf{b}$ into account, this vector equation can be represented by the following three scalar equations

$$\begin{cases} \int_S \mu dS = 1, \\ \int_S \eta dS = 0, \\ \int_S \gamma dS = 0, \end{cases} \tag{26}$$

thus placing restrictions on the unknown scalar functions (i.e., μ, η and γ).

Next, let us consider the requirement that the resultant moment produced by the continuum traction about its centre of mass (see *SI* Eq.(5)) has to be the same as that generated by the load $\mathbf{f}_j$, which gives

$$\int_S \boldsymbol{\rho} \times \mathbf{t}^{(n)} dS = \boldsymbol{\rho}_j \times \mathbf{f}_j. \tag{27}$$

Substitution of *SI* Eq.(23) into this relation results in

$$\left(\int_S \eta\boldsymbol{\rho} dS\right) \times \mathbf{a} + \left(\int_S \gamma\boldsymbol{\rho} dS\right) \times \mathbf{b} = \left[\boldsymbol{\rho}_j - \left(\int_S \mu\boldsymbol{\rho} dS\right)\right] \times \mathbf{f}_j. \tag{28}$$

Finally, let us examine the requirement that the power produced by the continuum traction (see *SI* Eq.(6)) has to be the same as that generated by the load $\mathbf{f}_j$, which gives

$$\int_S \tilde{\mathbf{v}} \cdot \mathbf{t}^{(n)} dS = \tilde{\mathbf{v}}_j \cdot \mathbf{f}_j. \tag{29}$$

It should be noted that the first order approximated particle velocity $\tilde{\mathbf{v}}_j$ (see *SI* Eq.(22)) is used here rather than the actual particle velocity $\mathbf{v}_j$. Substitution of *SI* Eq.(23) into this relation leads

to

$$\mathbf{L}:\left[\mathbf{a}\otimes\left(\int_S \eta\boldsymbol{\rho}dS\right)\right]+\mathbf{L}:\left[\mathbf{b}\otimes\left(\int_S \gamma\boldsymbol{\rho}dS\right)\right]=\mathbf{L}:\left\{\mathbf{f}_j\otimes\left[\boldsymbol{\rho}_j-\left(\int_S \mu\boldsymbol{\rho}dS\right)\right]\right\}. \tag{30}$$

As this should be satisfied for any possible $\mathbf{L}$, one has

$$\begin{cases} \boldsymbol{\rho}_j-\left(\int_S \mu\boldsymbol{\rho}dS\right)=\mathbf{0}, \\ \int_S \eta\boldsymbol{\rho}dS=\mathbf{0}, \\ \int_S \gamma\boldsymbol{\rho}dS=\mathbf{0}. \end{cases} \tag{31}$$

The conditions placed by requiring the same resultant force (see *SI* Eq.(26)), moment (see *SI* Eq.(28)) and power (see *SI* Eq.(31)) can be simultaneously satisfied by the following selections

$$\begin{cases} \mu=\frac{1}{V}\left(\frac{1}{3}\boldsymbol{\rho}+\boldsymbol{\rho}_j\right)\cdot\mathbf{n}, \\ \eta=0, \\ \gamma=0. \end{cases} \tag{32}$$

After substituting these functions into *SI* Eq.(23), the continuum traction $\mathbf{t}^{(n)}$ is thus given by

$$\mathbf{t}^{(n)}=\left[\frac{1}{V}\left(\frac{1}{3}\boldsymbol{\rho}+\boldsymbol{\rho}_j\right)\cdot\mathbf{n}\right]\mathbf{f}_j. \tag{33}$$

A stress tensor related to this traction can then be defined by either Cauchy's theorem $\mathbf{t}^{(n)}=\mathbf{n}\boldsymbol{\sigma}$ or $\mathbf{t}^{(n)}=\bar{\boldsymbol{\sigma}}\mathbf{n}$ depending on the chosen convention. The former relation leads to a continuum stress $\boldsymbol{\sigma}$ given by

$$\boldsymbol{\sigma}=\frac{1}{V}\left(\frac{1}{3}\boldsymbol{\rho}+\boldsymbol{\rho}_j\right)\otimes\mathbf{f}_j, \tag{34}$$

and the latter results in a continuum stress $\bar{\boldsymbol{\sigma}}$ given by

$$\bar{\boldsymbol{\sigma}}=\frac{1}{V}\mathbf{f}_j\otimes\left(\frac{1}{3}\boldsymbol{\rho}+\boldsymbol{\rho}_j\right). \tag{35}$$

It should be noted that both stresses are linear in terms of the position $\boldsymbol{\rho}$ and the particle force $\mathbf{f}_j$. In addition, it must be emphasised that unlike the Cauchy stress, both definitions imply a non-symmetric stress. In the following the first definition of stress is adopted.

The volume-averaged stress $\boldsymbol{\sigma}_V$ can be defined in a manner similar to that in continuum me-

chanics as

$$\boldsymbol{\sigma}_V = \frac{1}{V}\int_V \boldsymbol{\sigma} dV. \tag{36}$$

After substituting *SI* Eq.(34) into this equation and noting that the centroid of volume of a homogeneous continuum body coincides with its centre of mass, i.e., $\int_V \boldsymbol{\rho} dV = \mathbf{0}$, the volume-averaged stress can now be written as

$$\boldsymbol{\sigma}_V = \frac{1}{V}\boldsymbol{\rho}_j \otimes \mathbf{f}_j. \tag{37}$$

Similarly, the surface-averaged stress $\boldsymbol{\sigma}_S$ can also be defined as

$$\boldsymbol{\sigma}_S = \frac{1}{S}\int_S \boldsymbol{\sigma} dS. \tag{38}$$

After substituting *SI* Eq.(34) into this equation, the surface-averaged continuum stress is thus given by

$$\boldsymbol{\sigma}_S = \frac{1}{3VS}\left(\int_S \boldsymbol{\rho} dS\right) \otimes \mathbf{f}_j + \frac{1}{V}\boldsymbol{\rho}_j \otimes \mathbf{f}_j, \tag{39}$$

where the surface integration $\int_S \boldsymbol{\rho} dS$ is often equal to zero for many basic solid shapes (e.g., ellipsoid, rectangular prism and elliptical cylinder) as a result of geometric symmetry.

2.5 Generalisation to many particles

This section focusses on applying the concept of continuum stress to a more complex system involving different types of forces exerted on many particles. To this end, the continuum stress field (see *SI* Eq.(34)) obtained for the case involving a single force is generalised via the superposition principle, as it is linear in terms of the particle force.

Let $\mathbf{f}_j$ and $\boldsymbol{\sigma}_j$ respectively denote the resultant of all the forces being considered that are applied on particle j ($j \in \Omega$) and its associated continuum stress. The resultant stress $\boldsymbol{\sigma}$ associated with the particle group Ω can be written as

$$\boldsymbol{\sigma} = \sum_{j\in\Omega} \boldsymbol{\sigma}_j, \tag{40}$$

where $\boldsymbol{\sigma}_j = \frac{1}{V}\left(\frac{1}{3}\boldsymbol{\rho} + \boldsymbol{\rho}_j\right) \otimes \mathbf{f}_j$ (no sum over j). As is commonly used in continuum mechanics (see Ref.[3]), the stress can be separated into the hydrostatic pressure p and the deviatoric stress $\mathbf{S}$ through the following relation

$$\boldsymbol{\sigma} = -p\mathbf{1} + \mathbf{S}, \tag{41}$$

where $p = -\frac{1}{3}tr(\boldsymbol{\sigma})$, giving insight into the possibility of volumetric compaction or expansion. The deviatoric stress $\mathbf{S}$ is defined as

$$\mathbf{S} = \boldsymbol{\sigma} + p\mathbf{1} \tag{42}$$

and provides information on shearing, for example, through the scalar von Mises stress σ_{vm} defined by

$$\sigma_{vm} = \sqrt{\frac{3}{2}}\sqrt{\mathbf{S} : \mathbf{S}}. \tag{43}$$

2.5.1 Internal stress of a group of interacting particles

For a set Ω^{int} of interacting particles, let $\mathbf{f}_j$ represent the resultant of the forces exerted on particle j ($j \in \Omega^{int}$) by all other particles within the same set. Let V_{int} (see *SI* Eq.(15)) denote the volume of the solid homogeneous ellipsoid equivalent to this particle set. The resultant continuum stress associated with set Ω^{int} is termed the internal stress and given by

$$\boldsymbol{\sigma} = \sum_{j \in \Omega^{int}} \left[\frac{1}{V_{int}} \left(\frac{1}{3}\boldsymbol{\rho} + \boldsymbol{\rho}_j \right) \otimes \mathbf{f}_j \right]. \tag{44}$$

As Newton's third law requires $\sum\limits_{j \in \Omega^{int}} \mathbf{f}_j = \mathbf{0}$, the internal stress for this set of particles becomes

$$\boldsymbol{\sigma} = \frac{1}{V_{int}} \sum_{j \in \Omega^{int}} (\boldsymbol{\rho}_j \otimes \mathbf{f}_j). \tag{45}$$

The surface-averaged internal stress can then be calculated from

$$\boldsymbol{\sigma}_S = \frac{1}{V_{int}} \sum_{j \in \Omega^{int}} (\boldsymbol{\rho}_j \otimes \mathbf{f}_j). \tag{46}$$

The internal stress can be used as a continuum-level measure of how particles within one group mechanically influence each other.

2.5.2 Interaction stress between two interacting groups

For two sets Ω_1 and Ω_2 of interacting particles, let $\mathbf{f}_j$ represent the resultant of the forces on particle j ($j \in \Omega_1$) that are exerted by all the particles in set Ω_2. Let V_1 (see *SI* Eq.(15)) denote the volume of the solid homogeneous ellipsoid equivalent to the particle set Ω_1. The associated resultant continuum stress is termed the interaction stress induced by the particles in set Ω_2 on the

particles in set Ω_1 and given by

$$\boldsymbol{\sigma} = \sum_{j \in \Omega_1} \left[\frac{1}{V_1} \left(\frac{1}{3} \boldsymbol{\rho} + \boldsymbol{\rho}_j \right) \otimes \mathbf{f}_j \right]. \tag{47}$$

The surface-averaged interaction stress can then be calculated from

$$\boldsymbol{\sigma}_S = \frac{1}{V_1} \sum_{j \in \Omega_1} (\boldsymbol{\rho}_j \otimes \mathbf{f}_j). \tag{48}$$

The interaction stress can be used as a continuum-level measure of how the discrete particle groups mechanically interact with each other.

References

[1] Robin O. Cleveland and James A. McAteer. *The Physics of Shock Wave Lithotripsy.* Smith's Textbook of Endourology, 2012.

[2] Dongli Li, Antonio Pellegrino, Andre Hallack, Nik Petrinic, Antoine Jerusalem, and Robin Cleveland. Charaterisation of single cell response to shock waves and in silico optimisation of shock wave-mediated therapy. *submitted*, x(x):x, x.

[3] Mehrdad Negahban. *The Mechanical and Thermodynamical Theory of Plasticity.* CRC Press, Taylor & Francis Group, 2012.

[4] Lili Zhang, John Jasa, George Gazonas, Antoine Jérusalem, and Mehrdad Negahban. Extracting continuum-like deformation and stress from molecular dynamics simulations. *Computer Methods in Applied Mechanics and Engineering*, 283(0):1010–1031, 2015.

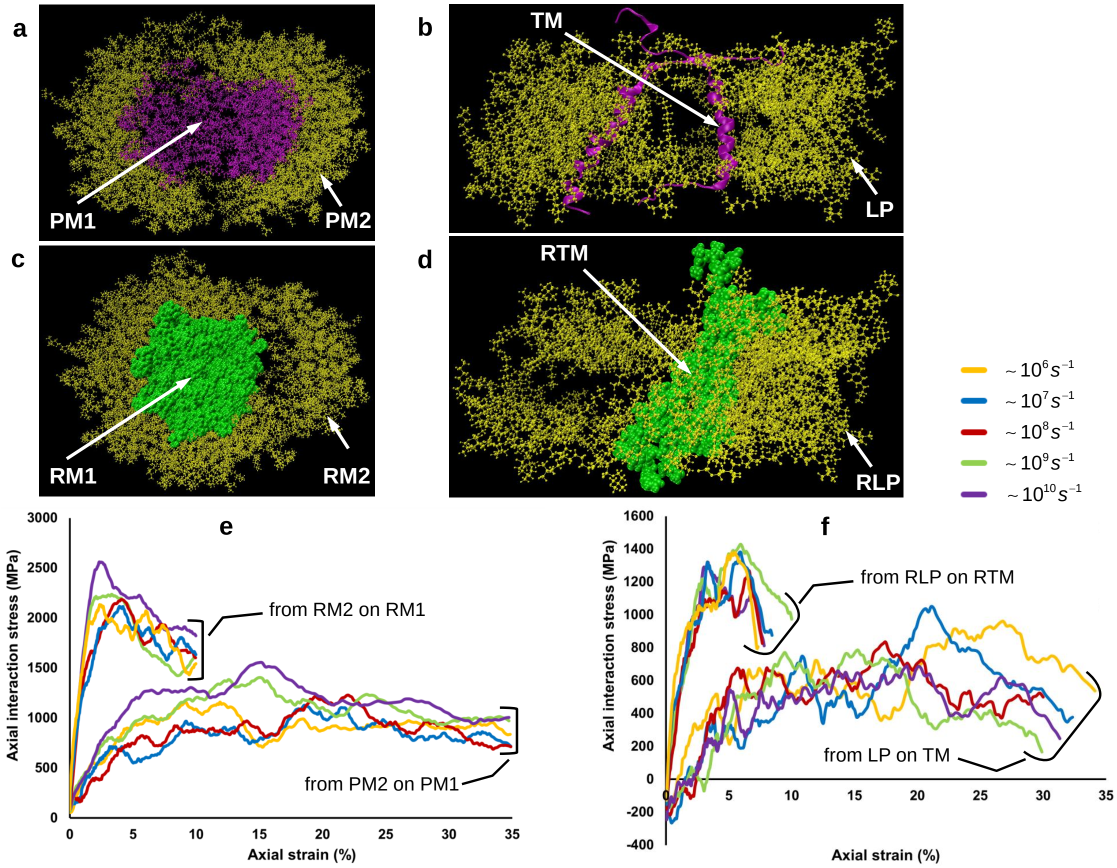

Figure 1: Highlighted representative structural alterations of the molecular groups in response to the uniaxial membrane stretch at $10^6 s^{-1}$, and the evolutions of surface-averaged interaction stresses with the membrane strains applied at various loading rates: (a) for the groups of pure lipid bilayer at membrane strain $\sim 50\%$; (b) for the groups of integrin-membrane complex at membrane strain $\sim 75\%$; (c) for the groups of lipid bilayer patch embedded with rigid lipid inclusions at membrane strain $\sim 20\%$; (d) for the groups of lipid bilayer patch embedded with rigid integrin inclusion at membrane strain $\sim 45\%$; and the evolutions of surface-averaged interaction stresses with the membrane strains along the axial loading direction at various loading rates for the groups of (e) pure lipid bilayer and its rigid counterpart and (f) integrin-membrane complex and its rigid counterpart.

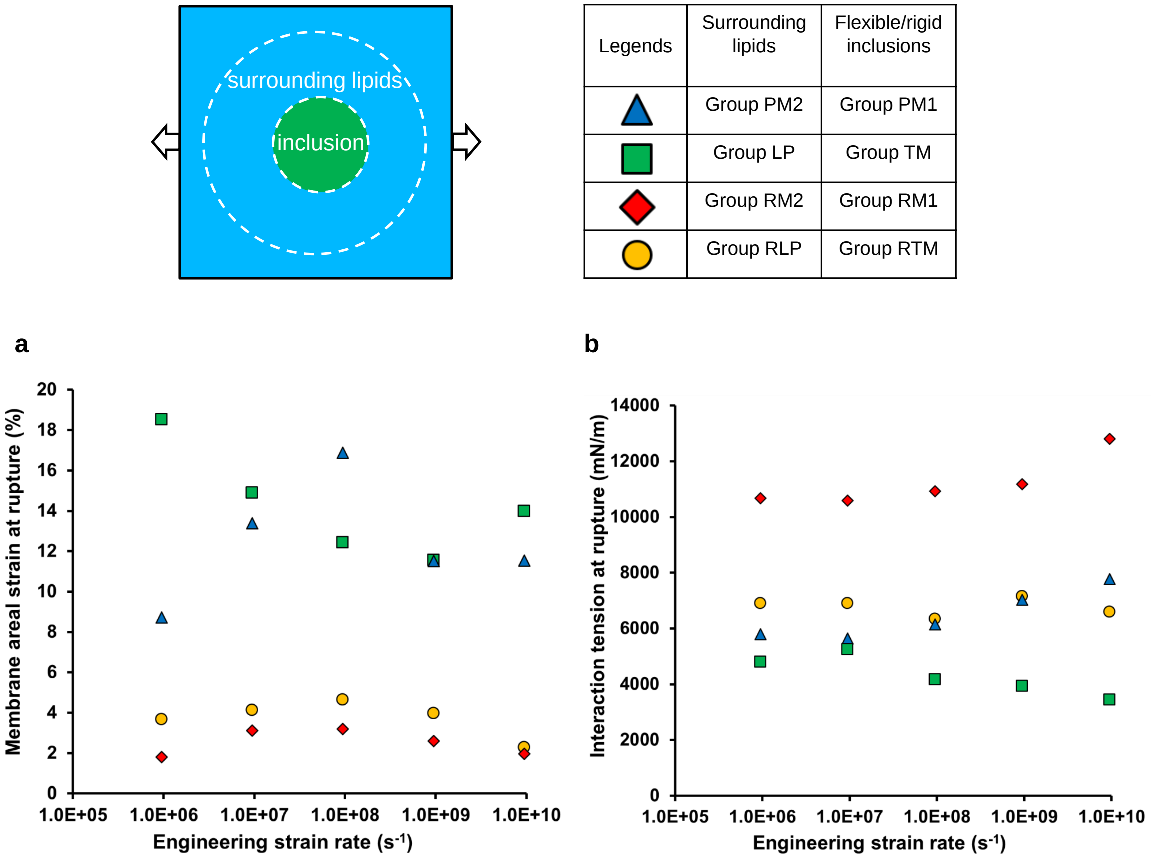

Figure 2: The continuum-level rupture characteristics of the chosen cellular membrane complexes under tension: (a) and (b) show the membrane areal strains at rupture and the corresponding rupture tensions estimated from the surface-averaged interaction stresses, respectively, for the biomolecular systems undergoing uniaxial membrane stretches (engineering strain rate range of $\sim 10^6 - 10^{10} s^{-1}$). The groups designated as the flexible/rigid inclusions and the associated surrounding lipids are listed: blue colour denotes the rupture characteristics involving groups PM1 and PM2 of the pure lipid bilayer membrane; green, red and orange colours represent the stretch-induced membrane complex rupture features for the integrin-membrane complex, the lipid bilayer embedded with rigid lipid inclusions and the lipid bilayer embedded with rigid integrin inclusion, respectively.

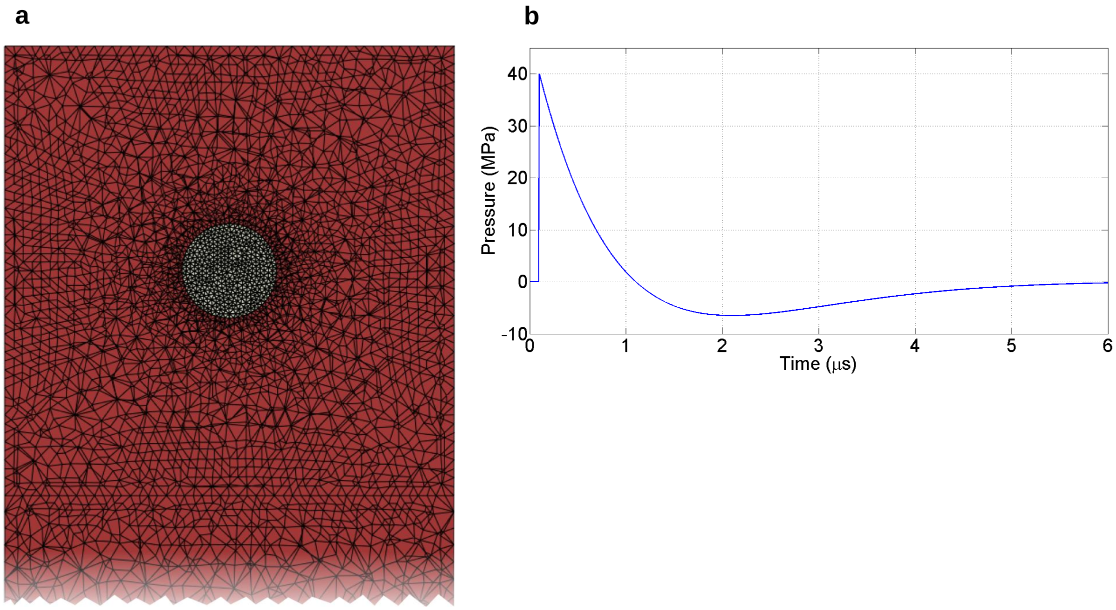

Figure 3: 3D FE simulation setup aimed at evaluating the whole-cell response to therapeutic shock waves. (a) Cross-sectional view from the mid-plane of the FE model: a whole cell with a differentiated cortical membrane (diameter: $\sim 20\ \mu m$; thickness: $200\ nm$) embedded in the surrounding tissue (adapted from Ref.[2]). (b) Full analytical shock wave profile representing a pressure wave measured at the focus of a clinical lithotripter [1].

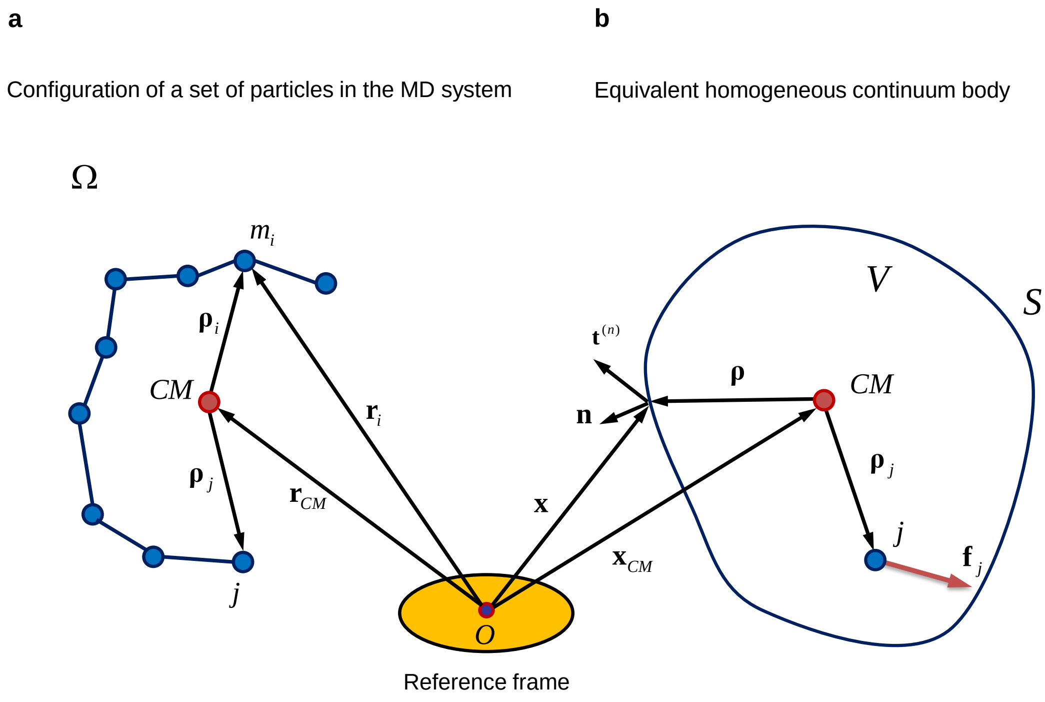

Figure 4: Schematic description of (a) a set of particles in the MD simulation and (b) a continuous traction distribution over the surface of an equivalent homogeneous continuum body, that produces the same resultant force, moment and power as the single force does on the particle.
